# Care of the Teeth

**Published:** 1892-06

**Authors:** 


### CARE OF THE TEETH.

The first requisite for keeping the teeth and gums sound and beautiful is to eschew the use of dentifrices of whose component ingredients nothing is known. An excellent recipe for a harmless powder to be made at home is the following:—Two ounces of prepared chalk, ½ oz. of pulverized white castile soap, 2 oz. of powdered orris-root, 2 tablespoonfulls of powdered sugar, a few drops of oil of wintergreen added as a flavoring. A powder of this sort can be used with safety twice each day. Some women use charcoal from time to time to whiten the teeth, but the practice cannot be recommended. Any rough or gritty substance naturally scratches the enamel, which becomes a repository in this abraded condition for tartar spots. The great point is to keep the enamel perfectly smooth. To remove the discolorations that will make their appearance on the best cared-for teeth use a small twig of althea, cherry, or other soft wood sharpened into a pencil point and rub the surface of the teeth, and the corners especially, with the same. Don't encourage yourself in brushing the teeth in warm water. Cold is a better tonic for the gums. Never brush sideways, which causes recession of the gums, but always with an up and down movement. And avoid, above all things, very stiff tooth-brushes and very large ones. The medium sizes, with soft bristles, are more healthy for the gums, and with the help of the wood pencils alluded to, will keep the teeth in perfect condition.—*Ex.*

